# Editorial: Neurosyphilis: epidemiology, clinical manifestations, diagnosis, immunology and treatment

**DOI:** 10.3389/fmed.2023.1191113

**Published:** 2023-04-19

**Authors:** Wujian Ke, Lai Sze Tso, Dongdong Li

**Affiliations:** ^1^Department of Sexually Transmitted Diseases, Dermatology Hospital of Southern Medical University, Guangzhou, Guangdong, China; ^2^Department of Sociology and Anthropology, Gustavus Adolphus College, Saint Peter, MN, United States; ^3^Division of Clinical Microbiology, Department of Laboratory Medicine, West China Hospital of Sichuan University, Chengdu, China

**Keywords:** neurosyphilis, *Treponema pallidum*, diagnositic, therapy, perspective

On 18 July 2022, the World Health Organization issued “*Global health sector strategies on HIV, viral hepatitis, and sexually transmitted infections, respectively, for the period of 2022 to 2030*” (1). This document highlights the pressing recommendation that early detection and treatment of sexually transmitted diseases should be strengthened to prevent serious complications or sequelae. Neurosyphilis, caused by *Treponema pallidum* (*Tp*), a frightening complication of syphilis, is a growing global concern. Challenges from neurosyphilis pathogenesis, diagnostic therapy, and prognosis remain obstacles to implementing effective prevention and control of neurosyphilis worldwide. In this Research Topic, we provide some viewpoints and discuss recent advancements to help accelerate scientific communication in the specialty of neurosyphilis.

To date, neurosyphilis is confirmed in laboratory settings via reactive results in non-treponemal (nTTs) and treponemal (TTs) serological tests, together with neurological or ophthalmological symptoms and cerebrospinal fluid (CSF) abnormalities, such as high WBCs, high protein concentrations, or positive VDRL or FTA-ABS test results (2–4). Although the performance of molecular assays is unsatisfactory, given that a negative PCR test may not rule out Tp infection, the application of assays serves as a potential supplementary tool for the diagnosis and differential diagnosis of neurosyphilis (5). This option requires consideration that CRISPR-based approaches may increase the clinical sensitivity (6) and Tp can be detected in CSF by metagenome sequencing (mNGS) in patients (7).

Serology is necessary for everyday clinical practice. NTTs exhibited a higher pooled specificity, and TTs exhibited a higher pooled sensitivity in diagnosing neurosyphilis on CSF (Xie et al.). However, the VDRL test remains the only nTTs that can be used in CSF samples. TRUST or PRP can be used as replacements due to the unavailable VDRL in China (8, 9). TRUST has a higher pooled sensitivity (0.83) than VDRL (0.77) and RPR (0.73) (Xie et al.). This sensitivity difference may be caused by different volume antigens used in nTTs as the manufacturer's instructions for the VDRL antigen test for diagnosing neurosyphilis describe testing of serum samples and do not include procedures for CSF testing. CSF-VDRL test with 17 μL of antigen instead of 10 μL was more sensitive (Xiao et al.). Beyond conventional biomarkers, some novel or potential laboratory indicators of neurosyphilis in CSF have reported effective results (10). Cytokine tests, macrophage migration inhibitory factor (MIF), IL-10, IL-17A, IL-26, and other biomarkers are demonstrating utility for the diagnosis of neurosyphilis (11). Among them, CXCL13 is used to evaluate the effectiveness of treatment for neurosyphilis (12). Some proteins indicating pathological damage in the central nervous system, sTREM2, neurofilament light chain (13), β2-microglobulin, tau protein, and BACE1 (Gao et al.), may also be potential indicators usedin diagnosing neurosyphilis but show low specificity and need further study (Gao et al.). All the above-mentioned biomarkers are associated with intrathecal immune response or injury related to *Tp*. The assessment of protein intrathecal synthesis (ITS) is an essential step in CSF analysis, especially pathogen-specific immunoglobin. The pattern of Ig ITS was IgIF-G (48.62%) > IgIF-A = IgIF-M (*P* < 0.05), with the dominant intrathecal fraction being IgG (median, 48.62%), which was also verified by Q_IgG_ > Q_alb_ > Q_IgM_ = Q_IgA_. As well, intrathecal IgM and IgG were associated with a parenchymatous type of neurosyphilis (Huang et al.). An intrathecal synthesis index of specific anti-*Treponema* IgG is a new promising tool highly specific for NS diagnosis (14). The formation of Ectopic germinal centers (EGCs) observed in the intracranial syphilitic gumma suggests aberrant humoral immune responses (15). Changes in T lymphocyte subsets (16) and functions (17, 18) in patients with neurosyphilis further indicate cellar immune responses. Hence, intrathecal response may be a future potential target for therapies or diagnoses to prevent persistent infection and disease progression. Some potential blood biomarkers for diagnosing neurosyphilis without requiring lumbar puncture are UCH-L1, GFAP, and NF-L (19). Also, serum TRSUT Titer ≥1: 16 is a predictor for neurosyphilis in HIV negative patients (20). The usefulness of novel biomarkers can further enhance the diagnostic sensitivity and specificity for neurosyphilis.

Tp -specific protein biomarkers can also be explored by mass spectrometry (21, 22). The roles of these proteins were explored, delving into the promotion of proinflammatory cytokine secretion (23) and cell migration (Tp0136), apoptosis (Tp0751), autophagy and adhesion (Tp768) (24–26), inducing angiogenesis (Tp47), and stimulating human platelet activation and aggregation (Tp0136) (27). This research facilitates the development of biomarker or vaccine candidates (Tp0954), especially in understanding neuropathogenesis. Neurosyphilis therapy options are limited, with aqueous penicillin being the first-line treatment. However, its short half-life requires intravenous infusion, prolonging hospital stays. A retrospective multicenter study in France concluded that ceftriaxone was as effective as benzylpenicillin, with a shorter hospital stay and fewer side effects (28). However, some authors raised concerns about the study's methodology, sample size, and potential selection bias, suggesting that more research is necessary to confirm the efficacy of ceftriaxone in treating neurosyphilis (29). Nonetheless, the study highlights ceftriaxone's potential as an alternative treatment for neurosyphilis.

To sum up, six articles are included in this Research Topic, three articles referring to diagnostic advances (Gao et al.; Xiao et al.; Xie et al.), one article highlighting the significance of intrathecal immune response (14), one article discussing Tp vaccine (He et al.), and a perspective proposing the CARE-NS research strategy to enhance the clinical management of neurosyphilis (Du et al.). We anticipated that this topic will provide powerful scientific evidences or some new thoughts for the prevention and control of neurosyphilis. Due to persistent challenges in diagnosing and treating Tp and its sequelae, neurosyphilis has become an ever-pressing debacle. In line with WHO recommendations, we call for more action to combat spirochetes and their infection. Undoubtedly, in the months and years to come, medicine will continue to expand upon this Research Topic.

## Author contributions

WJK and DDL wrote the manuscript. LST edited the manuscript. DDL did the final checks of the manuscript and submitted it. All authors contributed to the article and approved the submitted version.
